# Supplementary material for: Polycyclic Aromatic Hydrocarbons Contamination of Flamed and Braised Chickens and Health Risk Assessment in Burkina Faso
Source: Toxics. 2021 Mar 18;9(3):65. doi: 10.3390/toxics9030065 (PMC8002855; doi:10.3390/toxics9030065)
Supplement: Supplementary file 1 [file toxics-09-00065-s001.pdf]

Supplementary Materials: Polycyclic Aromatic Hydrocarbons Contamination of Flamed and Braised Chickens and Health Risk Assessment in Burkina Faso

Bazoin Sylvain Raoul Bazié, Caroline Douny, Thomas Judicaël Ouilly, Djidjoho Joseph Hounhouigan, Aly Savadogo, Elie Kabré, Marie-Louise Scippo and Imaël Henri Nestor Bassole

Questionnaire S1. Questionnaire for consumers of processed chickens.

QUESTIONNAIRE FOR CONSUMERS OF PROCESSED CHICKENS<br>*2016 - UJKZ/QUALISANI*			
General Information			
	1. Date/Number of the sheet		
	2. Code<br>3. District number<br>4. Sector number<br>5. Neighbourhood name		
Consumer identification			
	6. Indicate the Surname and First Name of the respondent		
	7. Gender O M O F<br>8. What is your profession ?<br>9. What is your level of education ?<br>O Primary O Secondary O Higher O Non-literate O Other O Autre O<br>10. How old are you?		
Consumption of processed chicken			

11. Do you know the following processed chicken? *(multiple responses possible)*
O Braised O Flamed O Smoked O Distant-grilled O Other……

12. For each type, how many times do you consume chicken on average per month? *(multiple responses possible)*
Braised…… Flamed ….. Smoked…… Distant-grilled….. Other…..

13. With how many personn do you eat chicken ? *(multiple responses possible)*
Braised…… Flamed ….. Smoked…… Distant-grilled….. Other…..

14.In what occasions do you eat processed chicken?? *(multiple responses possible)*

O Holidays, O ordinary days

15. Where do you eat the processed chicken? *(multiple responses possible)*

O Home O Restaurant (Bar)

16. What types of dishes do you eat with the processed chicken

Perception of quality

18. In your opinion, what are the quality criteria for a good braised? Why ?

18. In your opinion, what are the quality criteria for a good flamed ? Why ?

18. In your opinion, what are the quality criteria for a good smocked chcicken? Why ?

20, In your opinion, what are the quality criteria for a good Distant-grilled chicken? Why?

21. What type of processed chicken do you prefer? *(multiple responses possible)*
O Braised O Flamed O Smoked O Distant-grilled O Other

22. What are your selection criteria when you buy Braised chicken'. *(multiple responses possible)*
0 Taste of the chicken 0 Site hygiene 0 Processing method 0 Other

23. What are your criteria for choosing Flamed chicken? *(multiple responses possible)*
0 Taste of the chicken 0 Site hygiene O Processing method O Other.

24. What criteria do you use when buying 'smoked chicken'? *(multiple responses possible)*
O Taste of the chicken O Site hygiene O Processing method O Other

25. What do you look for when you buy the Distant-grilled chicken? *(multiple responses possible)*
O Taste of the chicken O Site hygiene O Processing method O Other

26. What are the quality criteria for which you are accepting to pay more

27. In what price range do you pay for chicken?
Braised……..Flamed.......Smoked..........Distant-grilled ....

28. Have you ever been sick after eating processed chicken'?
O Yes O No

29, If Yes, what type of chicken made you sick?
O Braised O Flamed O Smoked O Distant-grilled O Other

30. Do you think that processed chicken would have any therapeutic effects or virtues.
O Yes O No O Don't know

31, If yes, what are the virtues?

32. If yes, what are the virtues?

33. Do you consume other types of grilled products?
If yes, which ones?
….
34. Do you consume other types of smoked products?
If yes, which ones
